# A Segmentation Scheme for Complex Neuronal Arbors and Application to Vibration Sensitive Neurons in the Honeybee Brain

**DOI:** 10.3389/fninf.2018.00061

**Published:** 2018-09-26

**Authors:** Hidetoshi Ikeno, Ajayrama Kumaraswamy, Kazuki Kai, Thomas Wachtler, Hiroyuki Ai

**Affiliations:** ^1^School of Human Science and Environment, University of Hyogo, Himeji, Japan; ^2^Department Biologie II, Ludwig-Maximilians-Universität München, Martinsried, Germany; ^3^Department of Earth System Science, Fukuoka University, Fukuoka, Japan

**Keywords:** complex neuron segmentation, reconstruction, image processing, insect brain, confocal laser scanning microscopic image

## Abstract

The morphology of a neuron is strongly related to its physiological properties, application of logical product and thus to information processing functions. Optical microscope images are widely used for extracting the structure of neurons. Although several approaches have been proposed to trace and extract complex neuronal structures from microscopy images, available methods remain prone to errors. In this study, we present a practical scheme for processing confocal microscope images and reconstructing neuronal structures. We evaluated this scheme using image data samples and associated “gold standard” reconstructions from the BigNeuron Project. In these samples, dendritic arbors belonging to multiple projection branches of the same neuron overlapped in space, making it difficult to automatically and accurately trace their structural connectivity. Our proposed scheme, which combines several software tools for image masking and filtering with an existing tool for dendritic segmentation and tracing, outperformed state-of-the-art automatic methods in reconstructing such neuron structures. For evaluating our scheme, we applied it to a honeybee local interneuron, DL-Int-1, which has complex arbors and is considered to be a critical neuron for encoding the distance information indicated in the waggle dance of the honeybee.

## Introduction

Neuronal morphology is strongly related to neural function; neuron arborization reflects the input and output regions, and morphological characteristics, such as branching pattern, length and thickness, are related to signal transmission properties. Changes of neuronal morphology have been observed in various nervous systems, especially as a consequence of development or experience (Jan and Jan, 2003; Grueber et al., 2005; Yasunaga et al., 2010; Luebke et al., 2015) and difference of brain regions (Jacobs and Scheibel, 2002). For cortical pyramidal neurons, detailed analyses among different brain regions and species relating morphological results of optical and electron microscopy, and response characteristics from electrophysiological experiments have been performed (Elston, 2003; Spruston, 2008; Luebke, 2017). Under such circumstances, neuron segmentation and modeling is an effective way for quantitatively evaluating morphological properties of neurons and can be shared among researchers as a common resource (Halavi et al., 2008).

Neuron morphology is most commonly extracted from image stacks that are obtained by scanning dye-filled neuron in the brain under confocal laser scanning microscopes (CLSM). Various software tools have been developed and applied on neuron images. For light microscopy and confocal imaging data, segmentation tools based on manual operations, such as Neurolucida, NeuroExplorer, have been effectively used for segmentation and parameterization of neuron morphology (Benavides-Piccione et al., 2006; Bianchi et al., 2013; Magliaro et al., 2017). Semi-automatic and automatic segmentation tools were developed and applied on electron microscopy images (Lang et al., 2011; Berning et al., 2015; Jones et al., 2015). To extract and trace neuronal structure from CLSM images, we developed the software SIGEN (Yamasaki et al., 2006; Minemoto et al., 2009) and applied it for segmentation of neurons in the insect brain. This program uses a simple algorithm called single seed distance transform, which works effectively for the segmentation of various types of neurons. We have previously applied SIGEN to reconstruct several neurons in the moth brain, which were collected into a database and were used for neuron morphological analyses and network simulations (Ikeno et al., 2012).

As is well known, automatic segmentation of neuronal structure in CLSM images is one of the most challenging themes in the biological image processing, and several projects encompassing multiple research labs have been undertaken to foster better algorithms (Acciai et al., 2016). The DIADEM challenge (Brown et al., 2011) was a competition held to raise awareness of this problem and spur development of automatic reconstruction algorithms. In this project, a metric program for comparing two digital morphological reconstructions was developed (Gillette et al., 2011) and continues to be a widely used measure. Recently, the BigNeuron project has been conducted to develop automatic segmentation methods from CLSM images (Peng et al., 2015). Although automatic neuronal segmentation and reconstruction has significantly advanced by these efforts, it is still difficult to correctly reconstruct complex structured neurons. Hence, it can be useful to combine automatic segmentation tools with manual operations for effective analysis of neuronal structure.

One of the interesting application for neuronal structure analysis is to investigate of the neural mechanisms for encoding and decoding the spatial information to the profitable flower, indicated by honeybee waggle dance (von Frisch, 1967). Honeybees use a unique temporal pattern of vibration pulses caused by wing beats for informing spatial information to their hive mates. Airborne vibrations are received by sensory neurons located in Johnston’s organ of the antenna of the receivers. These sensory neurons mainly project their neurites into the dorsal lobes (DLs) in the brain (Ai et al., 2007). Among several types of interneurons which are sensitive to vibratory stimuli to the antenna (Ai et al., 2009, 2017), DL-Int-1 is a major local interneuron arborizing in the DL (Ai et al., 2009). This neuron shows tonic inhibitory responses during a train of vibratory stimuli applied to the antenna which has the same temporal structure as that received by receivers during waggle dance, suggesting the DL-Int-1 is one candidate for encoding the distance information to the location of flower indicated by waggle dancer (Ai et al., 2017). The soma is located close to the dorsal central body in the posterior protocerebral lobe (PPL), and a primary neurite extends into the DL. In the DL, two thick branches, called dorsal branch (DB) and ventral branch (VB), emerge from the primary neurite (Ai et al., 2009). These two branches overlap densely in the DL (Ai, 2010), which makes it difficult to separate them by any automatic segmentation process. To make a segmentation of the morphology of this interneuron, we have developed and applied a practical scheme, which combines a manual extraction and mask filtering of neuronal branches with an automatic segmentation by using SIGEN (Minemoto et al., 2009). In this study, we evaluated its performance by applying our new scheme to fruitfly interneurons and honeybee DL-Int-1 and found out that our new scheme are useful in segmentation of the neurons of which several arborizations overlap in the same region.

## Material and Methods

### Neuronal Segmentation and its Evaluation

**Figure 1:**
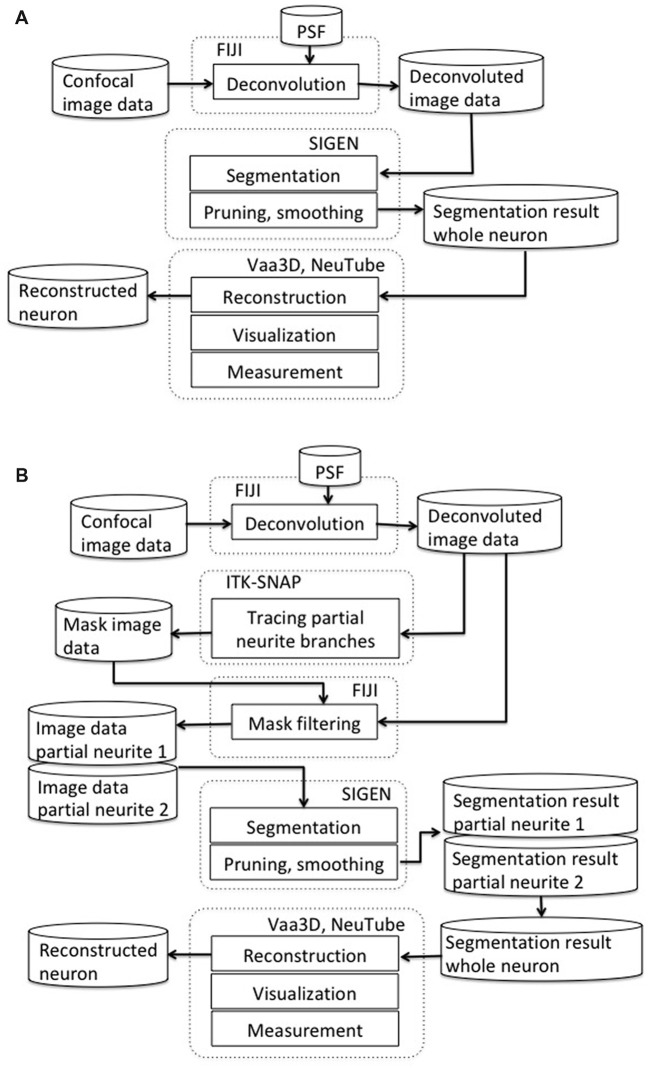
**(A)** Our basic neuron segmentation scheme from a confocal laser scanning microscopes (CLSM) image. The CLSM image was deconvolved by image processing software. Neuronal branches were extracted by the neuron segmentation software SIGEN and stored in SWC format. **(B)** Our revised segmentation scheme incorporating a process for manually separating overlapping neurites. After the CLSM image was deconvolved by image processing software, masked image data were generated by tracing partial neuronal branches on the deconvolved image data. A partial branch image was obtained by applying AND image operation between the deconvolved image and the mask image. Another part of the neuronal branch image was generated by exclusive-or (EOR) image operation between the deconvolved image and the partial branch image. Neuronal segmentation software was applied on these two branch images independently. Whole neuron structure was reconstructed by combining these two reconstruction results.

Our scheme for segmentation of CLSM image starts with deconvolution by Fiji (RRID: SCR_002285; Figure 1A). We used the “Iterative Deconvolve 3D” plugin along with the point spread function (PSF) that was generated by the “Diffraction PSF 3D” plugin with default parameter values (Dougherty, 2005). This is an effective process for decreasing blurring and distortion in CLSM images. Next, we conducted neuron segmentation by SIGEN (RRID: SCR_016284), which was applied to the deconvolved images. Segmentation results were obtained in SWC format, which is widely used as a standard data format to describe neuronal structure (Ascoli et al., 2007).

To evaluate the performance of our scheme based on SIGEN, we compared segmentation results with those of other available segmentation tools. The BigNeuron project^1^ provides a huge collection of image data containing singly stained neurons along with corresponding gold standard segmentation results. The gold standard results were manually segmented from image data and were used as reference morphologies when comparing different algorithms. We used 40 samples from the package “p_changed7_janelia_flylight_part1,” which includes neurons of various shapes from fly brain imaged using CLSM, as in our experiments. Parameter values for segmentation by SIGEN were as follows. Only those fragmented segments with volumes larger than 30 voxels were included in the segmentation (VT = 30). Further, fragmented segments whose distance from the main branch, after rounding to the nearest voxel, was less than 30 voxels (DT = 30), were connected to the reconstructed neuron. Segmentation results were evaluated using the DIADEM metric value (Gillette et al., 2011). DIADEM metric was developed and provided by the DIADEM Challenge project for evaluation of neuronal segmentation results by comparison to gold standard reconstructions. The DIADEM metric value ranges from 0 to 1 depending on the degree of matching of several features, such as node positions and branching state, with a value of 1 indicating a perfect matching.

Metric values of segmentation results of an algorithm can vary largely depending on the morphological complexity of input image data. In particular, when multiple neuronal branches spread their dendrites into the same region, it can be extremely difficult to accurately extract their structural connectivity by automatic segmentation. To apply our segmentation software to such complicated neuronal structures, we propose a revised scheme incorporating a process for manually separating overlapping neurites (Figure 1B). In our newly revised segmentation scheme, we traced the neurites manually to create a mask image for separating branches extending in the same region. For manual tracing, we used the 3D segmentation software, ITK-SNAP (Yushkevich et al., 2006; RRID:SCR_002010). The manual tracing operation started from a clearly separated neuronal branch point. Neuronal segments were connected with the branch point and its connected terminals by characteristics of the image, such as direction of intensity changes in a voxel cluster and characteristics of connectivity and bifurcation from the start point to peripherals. Since this process requires a certain level of skill, it causes a possibility of human error. However, the manual tracing of the partial neuronal branches often provides more reliable separation than the automatic tracing. Then, a partial neuronal image containing one of the overlapping branches was obtained by application of logical product (AND) image operation between the manual traced branching image and the deconvoluted neuron image. The image of the other overlapping branch was obtained by exclusive-or (EOR) image operation between the deconvoluted neuron image and the previously obtained partial neuron image. Since the EOR image operation results in the background value (0) when the values of voxels of the two images are the same, an image excluding the branches previously obtained from the original image is obtained. For the case where more neurites are densely concentrated in one region, the same procedure can be recursively applied by separating neurites into individual partial structures at each step.

The whole branching structure was obtained by conjunction of segmentation results of partial neurites. Finally, the segmented neuron structure was stored by the software in SWC and VTK formats. Thus, the results could be displayed and edited by other tools such as Vaa3D (Peng et al., 2010; RRID:SCR_002609), NeuTube (Feng et al., 2015), or ParaView (Ayachit, 2015; RRID:SCR_002516).

### Acquiring the CLSM Image of DL-Int-1 and its Segmentation

Neuron images of DL-Int-1 in the honeybee brain were obtained experimentally. The details of intracellular recording and staining procedures were described in Ai et al. (2009, 2017) and we describe them here briefly. Honeybees (*Apis mellifera L*.) were reared in hives at the Fukuoka University campus. Forager of worker bees, which collected pollen on their hind legs, were caught at the hive entrance and were used in this study. In addition, newly emerged juveniles were isolated in a cage when they hatched from brood cells. Their ages were recorded in days, and those less than 3 days old were used in our experiments. For the experiment, a bee was anesthetized by cooling and was mounted in an acrylic chamber. The bee was fed with 1 M sucrose solution and kept overnight in the dark with high humidity at 20°C. The head of the bee was fixed with wax. The frontal surface of the brain was exposed by cutting away a small rectangular window between the compound eyes. The glands and tracheal sheaths on top of the brain were removed. The mouthparts, including the mandibles, were cut off to expose and remove the esophagus. Small droplets of a honeybee physiological saline were applied to wash away the residue of the esophagus and to enhance electrical contact with a platinum ground electrode placed in the head capsule next to the brain.

Glass electrodes were filled at the tip with 3% Lucifer Yellow CH Dilithium salt (L0529, Sigma-Aldrich, Tokyo, Japan) dissolved in 100 mM KCl, with Dextran, Tetramethylrhodamine, 3000 MW, Anionic, Lysine Fixable (D3308, Thermo Fisher), or with Alexa 647 hydrazide (A20502, Thermo Fisher, Tokyo, Japan) yielding DC resistances in the range of 150–300 MΩ. The electrode was inserted into a region of the DL after the neuronal sheath and a small area of the brain’s neurilemma had been scratched. Electrical signals were amplified with an amplifier (MEZ 8301, Nihon Kohden, Tokyo, Japan) and displayed on an oscilloscope. After identifying DL-Int-1 by their unique response patterns to the vibration stimuli applied to the antenna, the fluorescent dyes were injected iontophoretically into the neuron.

Thereafter, the brains were removed from the head capsule, fixed in 4% paraformaldehyde for 4 h at room temperature, and then rinsed in phosphate buffer solution, dehydrated and cleared in methyl salicylate for subsequent observation. The cleared specimens containing intracellularly stained neurons were viewed from the posterior side of the brain under a CLSM (LSM 510, Carl Zeiss, Jena, Germany) with a Zeiss Plan-Apochromat 25×/0.8 NA oil lens objective (working distance 0.57 mm). Alexa 647 was excited by the 633-nm line of HeNe lasers, Dextran, Tetramethylrhodamine by 543-nm line of HeNe lasers and Lucifer yellow by the 488-nm line of an argon laser. More than 250 optical sections were taken at 1 μm thickness throughout the entire brain depth of each specimen. The image resolution of each section was 1024 × 1024 pixels and each pixel size covered 0.36 μm × 0.36 μm in square.

## Results

### Evaluation of Semiautomatic Segmentation Scheme

**Figure 2:**
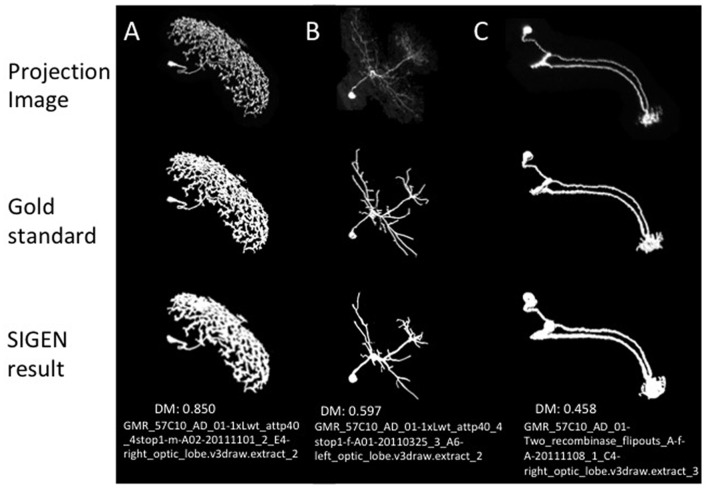
Three examples of segmentation by our original software, SIGEN, on samples provided by the BigNeuron project. *Top row*, 2D projected image data of neurons. *Middle row*, the manually segmented gold standard results, provided by BigNeuron. *Bottom row*, segmentation results by SIGEN. The difference between gold standard and automatic segmentation results was measured by the DIADEM metric, whose value ranges from 0 (completely unmatched) to 1 (perfectly matched). A neuron with a complex branching structure was reconstructed well, as shown in sample (**A**; DIADEM value: 0.850). Even from the low contrast image, neuronal structure was reconstructed with relatively well (**B**; DIADEM value: 0.597). In contrast, the neuron shown in sample **(C)** was reconstructed with a low DIADEM metric value of 0.458, due to the presence of many tiny neurites that were difficult to be extracted, which was also seen in the gold standard results.

**Figure 3:**
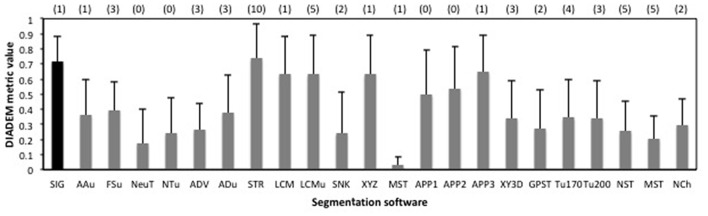
The average values of the DIADEM metric for 40 samples in the package “p_changed7_janelia_flylight_part1” of BigNeuron. Error bars indicate standard deviation, and the numbers in parentheses at the top of the graph are the number of samples for which no DIADEM value was obtained for the segmentation result. Abbreviations are segmentation software names: SIG, SIGEN; AAu, axis analyzer updated; FSu, fastmarching spanningtree updated; NeuT, neuTube; NTu, neuTube updated; ADV, Advantra; ADu, Advantra updated; STR, smartTracing; LCM, LCMboost; LCMu, LCMboost updated; SNK, snake; XYZ, All-path-pruning; MST, MOST; APP1, All-path-pruning1; APP2, All-path-pruning2; APP3, All-path-pruning3; XY3D, XY_3D_TreMap; GPST, NeuroGPSTree; Tu170, Tubularity model_S MST tracing th170; Tu200, Tubularity model S MST tracing th 200; NST, NeuroStalker; MST, MST Tracing; NCh, NeuronChaser updated.

To confirm the performance of our basic segmentation tool SIGEN, we applied it on samples provided by the BigNeuron project^2^. Neuron images with gold standard segmentation data can be downloaded from the site. We used samples in the subset “p_packed7_janelia_flylight-part1,” of the gold166 package. A neuron with complex structure was reconstructed well by SIGEN, resulting in a DIADEM metric value of 0.850 (Figure 2A). Even from the low contrast image, neuronal structure was reconstructed with relatively high DIADEM metric value of 0.597 (Figure 2B). In contrast, the metric value was relatively low for a neuron with simpler structure (Figure 2C). In this case, although thick and long branches were reconstructed well, SIGEN had difficulty tracing small dense neurites located in the lower right part of the image. However, experts also had difficulty manually tracing these dense arborizations, as seen from the gold standard results. The average value of the DIADEM metric value of SIGEN for 40 samples was 0.717 ± 0.163. Higher average values compared with other software and small values of variance indicate that neuronal morphology can be reconstructed stably by SIGEN (Figure 3). This package contains images of various shapes of insect neurons. Because SIGEN was able to successfully handle such neuron images, the software is considered to be suitable for the reconstruction of neuronal morphology.

**Figure 4:**
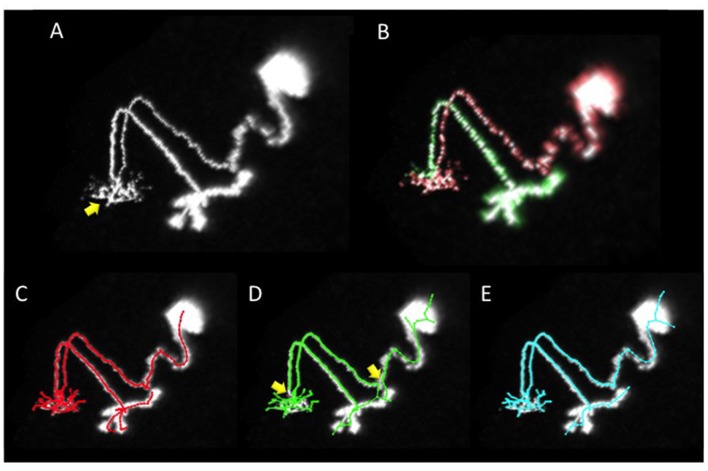
An example of segmentation of a neuron with several arborizations spatially overlapping. **(A)** Projection image of the neuron. The primary neurite from the soma extends and arborizes in the region indicated by the yellow arrow. A secondary neurite runs parallel with the primary neurite. **(B)** Two neurites (the soma and the primary neurite are shown in red, and the secondary neurite in green) were separated by manual operation in our revised scheme. **(C)** The gold standard of segmentation. **(D)** Segmentation result by our basic scheme (Figure 1A). **(E)** Segmentation result by our revised scheme (Figure 1B). In the result of our basic scheme in **(D)** there are segmentation errors on major connecting points of neurites (yellow arrows in **D**) most of which were rectified in the revised scheme in (**E**).

In the image set used above, some of the samples have multiple neurites mixed in one region. In such cases, all software approaches failed to extract good reconstructions. For example, the neuron “GMR_57C10_AD_01-Two_recombinase_flipouts_A-f-A-20111108_1_C4-right_optic_lobe.v3draw.extract_3” has a primary neurite extending from the cell body with neurites spreading in a certain region (arrow in Figure 4A). Furthermore, a secondary neurite from the dendrite runs along the primary neurite (Figures 4A,B). It is important to grasp the state of extension of such a neuron accurately, and in the gold standard by manual extraction, the primary neurite folded near the secondary neurite was accurately extracted (Figure 4C). However, in the reconstruction generated from our automatic segmentation software SIGEN (Figure 1A), the halfway and terminal points of the neurites were erroneously connected (compare Figures 4C,D yellow arrows). The structure was significantly different from the gold standard, resulting in a DIADEM metric value of 0.488. The wrong interpretation of the neurite form was not limited to SIGEN. The DIADEM metric values for other automatic segmentation software were comparatively low, with a maximum of 0.544 and an average of 0.151. By applying our revised segmentation scheme (Figure 1B), neurites elongating in different directions were separated and extracted, and then synthesized (shown in red and green in Figure 4B). It became possible to accurately extract the major cell structure, as shown by the large improvement of DIADEM metric value from 0.488 to 0.825 (Figure 4E). Therefore, for cases where multiple dendrites extend to the same area, our proposed method is more effective than all compared automatic segmentation methods. It is important to grasp the principal connection state of the neurites in constructing highly accurate morphological models and obtaining the characteristics of neurons. For complex branching structures, it is necessary to acquire knowledge about the connection in this part by a detailed analysis, such as tracing on locally acquired high resolution images. The information can be utilized for separation of major branched and has important meaning for understanding the structure of the whole neuron.

### Segmentation of a Vibration Sensitive Interneuron in Honeybee Brain

**Figure 5:**
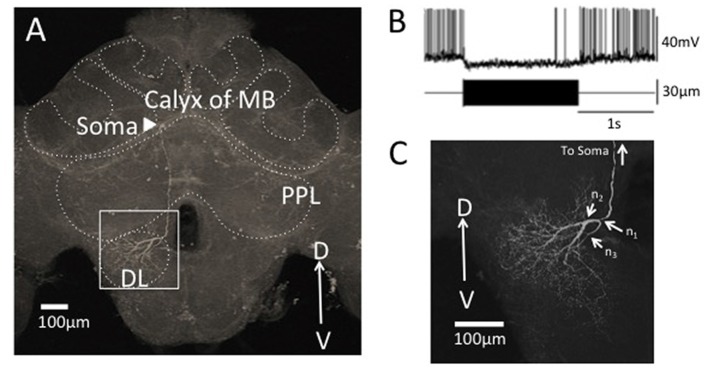
Segmentation of a vibration sensitive interneuron in the honeybee brain, DL-Int-1. **(A)** The whole morphology of DL-Int-1 in the honeybee brain. The soma (arrowhead) is located below the calyx of mushroom body (MB). A long primary neurite extends ventrally through posterior protocerebral lobe (PPL) and arborizes in the dorsal lobe (DL). **(B)** Typical unique electrophysiological response pattern of DL-Int-1. Tonic inhibitory response was observed by a continuous 265 Hz vibration stimulus to the antenna. The upper trace shows the action potentials of the DL-Int-1 (bottom trace: vibration stimulus). **(C)** Arborization of neurites of DL-Int-1 in the dorsal lobe. n_1_ is the point at which the primary neurite bifurcated into the dorsal branch (DB) and ventral branch (VB), which are difficult to be spatially separated. The DB started from the branching point n_2_. The VB started from the branching point n_3_.

In this study, we focused on the morphology of DL-Int-1 arborizations of DB and VB in the DL (n_2_ and n_3_ in Figure 5C, respectively). To obtain the whole arborizing image, separately captured CLSM image stacks were stitched in 3D space using Fiji (Preibisch et al., 2009; Schindelin et al., 2012).

**Figure 6:**
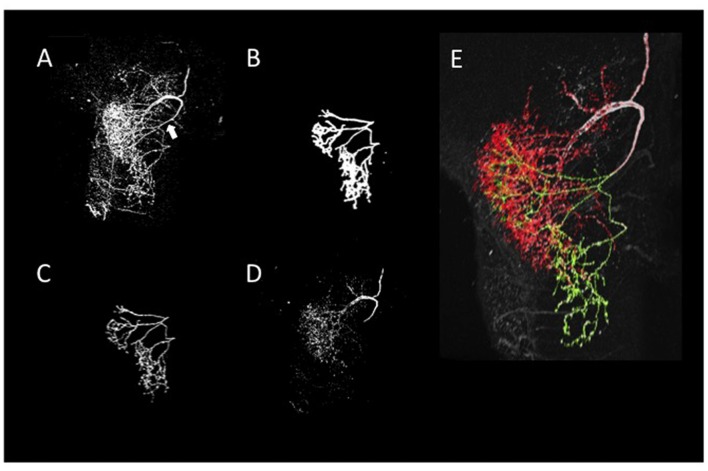
Reconstruction of neurite arborizations of DL-Int-1 by using our revised segmentation scheme. **(A)** 2D projection image of DL-Int-1 in the dorsal lobe. The white arrow indicates the point that SIGEN could not extract the correct connection by our basic scheme. **(B)** 2D projection image of a manually traced 3D mask filter for separation of VB and DB. **(C)** The extraction result of the VBs obtained by application of the mask filter **(B)** on the whole arborization image **(A)**. **(D)** The DBs obtained by the EOR image calculation between whole branching **(A)** and VBs **(C)**. **(E)** Skeleton traces of reconstruction overlapped with the original 2D-projected image. Green and red skeleton traces show VB and DB, respectively.

**Figure 7:**
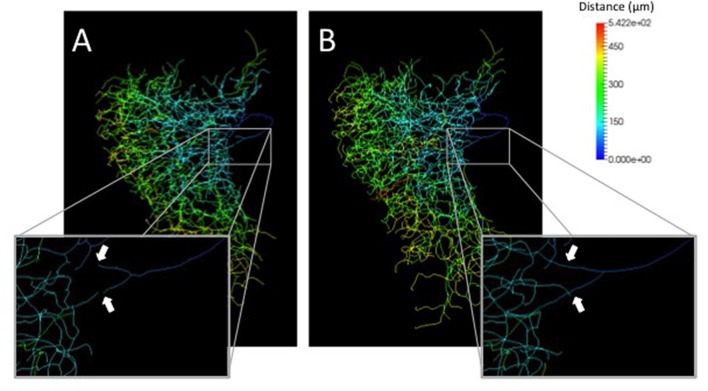
Comparison of distances from the root point on segmentation results by our basic **(A)** and revised **(B)** schemes. The distance from root points were indicated by pseudocolors. The enlarged views show locations where the connection is different between by the basic scheme and by our revised scheme (white arrows).

**Figure 8:**
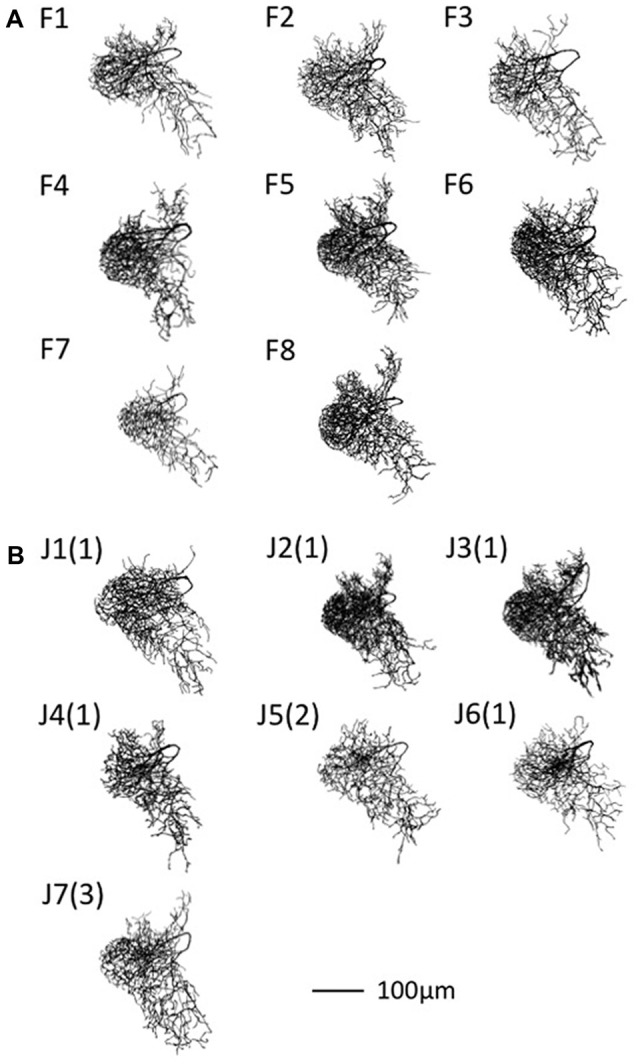
Segmentation results of DL-Int-1 for adult worker. **(A)** Forager (F). **(B)** Age-controlled juvenile (J). The following numbers of F and J indicate the sample index number. In **(B)** the numbers in parenthesis indicate the age in days.

A neurite terminal was automatically selected as the start segment (root point) of segmentation by SIGEN. After obtaining the neuronal structure by our segmentation tool, we changed the root point on the branching node of the neurite (n_1_ in Figure 5C) toward the soma. Shapes of neuronal branches were extracted well, but several connections among the branch segments were incorrect even at the major branching points, which connect to thick branches (white arrow in Figure 6A). To reconstruct the major neuronal structure correctly, we applied our revised segmentation scheme (Figure 1B). We generated a mask image for extracting the VB (Figure 6B) from the whole neuron image. The manually-traced mask image of VB was applied by AND image operation on the deconvoluted image to obtain the VB image (Figure 6C). The DB image was extracted by EOR image operation between the deconvoluted image and the VB image (Figure 6D). Segmentation and reconstruction of neuron morphologies were generated from these VB and DB images independently. The whole neuronal structure was reconstructed by connection of these two reconstructed results (Figure 6E). Although the overall shape of neuronal branches obtained by the automatical (Figure 1A) and our revised schemes (Figure 1B) was similar, connections of branches and distance from the root (start) point were quite different between the methods (Figure 7). As shown in the enlarged view, the thick VB neurite bifurcating into two neurites. One branch extends to the dorsal side and the other extends to the ventral side and branches again. Such a structure could be extracted correctly, as shown in Figure 7B, because the revised scheme had grasped the whole structure in advance. In contrast, it could not be extracted by SIGEN alone, and it was disconnected just after branching (Figure 7A). By our revised segmentation scheme, we obtained reconstruction results from eight foragers (F; Figure 8A) and seven age-controlled juveniles (J; Figure 8B). Detailed analyses based on morphometric features, such as branching patterns, branch segment length and diameter, could be addressed in future research to assess morphological differences between ages.

## Discussion

We applied the segmentation software SIGEN to CLSM images for reconstructing morphological models of neurons. It achieved high scores on benchmark tests of the BigNeuron samples. Its segmentation results demonstrated its reliability in extracting the major structure of a neuron without human bias. Various approaches to develop automatic and semi-automatic neuron segmentation software are being undertaken in fields including neuromorphometric research and neural computation (Lu et al., 2009; Sümbül et al., 2016; Li et al., 2017).

In this study, we also developed a segmentation scheme for neurons with complex arborizing patterns that performed better than the compared existing approaches. Preprocessing CLSM images was useful to separate the neuron image from the background. The main difficulty in extracting structures like that of DL-Int-1 was caused by multiple arborizations extending and overlapping in the same region. Hence, it was difficult to identify and connect them from fragmented images. We applied state-of-the-art automatic segmentation tools, such as App2 and SmartTracing (Chen et al., 2015), on these neurons. The reconstruction result involved many erroneous branches caused by noise and the background brain image. Although our revised scheme requires a user to conduct manual tracing to apply a mask filter, it produced more accurate segmentation results for structurally complex neurons. Manual tracing can introduce a problem with reproducibility, but at the present time it provides a more accurate result than fully automated techniques. Moreover, in our scheme the manual step is represented by the mask filter, which can be saved as objective documentation of the intervention.

Regarding the extraction of neurites, first, staining method and image quality have a great influence, and it was shown that more accurate morphology can result from images acquired by optical microscopy for specimens treated by DAB (Elston, 2007; Elston and Fujita, 2014). Comparison and evaluation with extraction results from confocal images acquired by confocal laser microscopy or two-photon microscopy are also important. Furthermore, the accuracy of extraction of morphology is highly dependent on the morphology of the neurons. Due to anisotropic resolution (usually the resolution in the Z direction is poor), extraction accuracy is often bad for a specific direction. Even in such a case, it is effective to acquire detailed connection information by manual segmentation, and reflect the result in the extraction of the whole neuron. In this respect, it could be effective to combine manual operation and automatic segmentation.

The DL-Int-1 interneuron is considered to play a key role in the primary auditory center for vibration signal processing. In this study, we have obtained more than 15 reconstructions of DL-Int-1 from foragers and newly emerged juveniles. Future research could apply detailed morphometric analyses to the segmentation results and evaluate the similarity and differences of neuronal morphologies with age.

## Conclusion

Automatic or semiautomatic segmentation techniques have become quite useful for morphometric analysis of neurons. In this study, we combined manual masking and filtering with an existing software for semi-automated dendritic segmentation of interneurons with complex structures. The efforts to develop segmentation techniques will benefit from new algorithms for image processing and machine learning in the future. However, this process is ongoing because segmentation results depend on various factors such as neuron form and image quality. Therefore, there is a continuing need to develop better automatic segmentation software tools. Until such a new tool is obtained, we think that it is best to respond flexibly by a combination of automatic segmentation software and manual operation, as proposed here. By using our developed procedure, we could proceed to analyze age- and labor-dependent morphometric change of critical interneuron related with deciphering dance communication in honeybee.

## Author Contributions

HA, TW and HI designed this work. KK and HA performed experiments to acquire neuron image data. HI and AK conducted segmentations. HI drafted the manuscript and all authors reviewed and approved the final version of manuscript.

## Conflict of Interest Statement

The authors declare that the research was conducted in the absence of any commercial or financial relationships that could be construed as a potential conflict of interest.

## Footnotes

^1^https://alleninstitute.org/bigneuron/

^2^https://github.com/BigNeuron/Data/releases
